# SWOT analysis of noninvasive tests for diagnosing NAFLD with severe fibrosis: an expert review by the JANIT Forum

**DOI:** 10.1007/s00535-022-01932-1

**Published:** 2022-12-05

**Authors:** Yoshihiro Kamada, Takahiro Nakamura, Satoko Isobe, Kumiko Hosono, Yukiko Suama, Yukie Ohtakaki, Arihito Nauchi, Naoto Yasuda, Soh Mitsuta, Kouichi Miura, Takuma Yamamoto, Tatsunori Hosono, Akihiro Yoshida, Ippei Kawanishi, Hideaki Fukushima, Masao Kinoshita, Atsushi Umeda, Yuichi Kinoshita, Kana Fukami, Toshio Miyawaki, Hideki Fujii, Yuichi Yoshida, Miwa Kawanaka, Hideyuki Hyogo, Asahiro Morishita, Hideki Hayashi, Hiroshi Tobita, Kengo Tomita, Tadashi Ikegami, Hirokazu Takahashi, Masato Yoneda, Dae Won Jun, Yoshio Sumida, Takeshi Okanoue, Atsushi Nakajima, JANIT Forum

**Affiliations:** 1 Department of Advanced Metabolic Hepatology, Osaka University Graduate School of Medicine, 1-7, Yamadaoka, Suita, Osaka, 565-0871 Japan; 2 Medicine Division, Nippon Boehringer Ingelheim Co., Ltd., 2-1-1, Osaki, Shinagawa-Ku, Tokyo, 141-6017 Japan; 3 FibroScan Division, Integral Corporation, 2-25-2, Kamiosaki, Shinagawa-Ku, Tokyo, 141-0021 Japan; 4 Immunology, Hepatology & Dermatology Medical Franchise Dept., Medical Division, Novartis Pharma K.K., 1-23-1, Toranomon, Minato-Ku, Tokyo, 105-6333 Japan; 5 Medical Information Services, Institute of Immunology Co., Ltd., 1-1-10, Koraku, Bunkyo-Ku, Tokyo, 112-0004 Japan; 6 Product Development 1St Group, Product Development Dept., Fujirebio Inc., 2-1-1, Nishishinjuku, Shinjuku-Ku, Tokyo, 163-0410 Japan; 7 Academic Department, GE Healthcare Japan, 4-7-127, Asahigaoka, Hino, Tokyo, 191-8503 Japan; 8 Ultrasound Business Area, Siemens Healthcare KK, 1-11-1, Osaki, Shinagawa-Ku, Tokyo, 141-8644 Japan; 9 Department of Medicine, Division of Gastroenterology, Jichi Medical University, 3311-1, Yakushiji, Shimotsuke, Tochigi, 329-0498 Japan; 10 Cardiovascular and Diabetes, Product Marketing Department, Kowa Company, Ltd., 3-4-10, Nihonbashi Honcho, Chuo-Ku, Tokyo, 103-0023 Japan; 11 Clinical Development & Operations Japan, Nippon Boehringer Ingelheim Co., Ltd., 2-1-1, Osaki, Shinagawa-Ku, Tokyo, 141-6017 Japan; 12 Medical Affairs Department, Kowa Company, Ltd., 3-4-14, Nihonbashi Honcho, Chuo-Ku, Tokyo, 103-8433 Japan; 13 R&D Planning Department, EA Pharma Co., Ltd., 2-1-1, Irifune, Chuo-Ku, Tokyo, 104-0042 Japan; 14 Diagnostics Business Area, Siemens Healthcare Diagnostics KK, 1-11-1, Osaki, Shinagawa-Ku, Tokyo, 141-8673 Japan; 15 Marketing Dep. H.U. Frontier, Inc., Shinjuku Mitsui Building, 2-1-1, Nishishinjuku, Shinjuku-Ku, Tokyo, 163-0408 Japan; 16 Clinical Development Dept, EA Pharma Co., Ltd., 2-1-1, Irifune, Chuo-Ku, Tokyo, 104-0042 Japan; 17 Global Drug Development Division, Novartis Pharma KK, 1-23-1, Toranomon, Minato-Ku, Tokyo, 105-6333 Japan; 18 2Nd Product Planning Dept, 2Nd Product Planning Division, Fujirebio Inc, 2-1-1, Nishishinjuku, Shinjuku-Ku, Tokyo, 163-0410 Japan; 19 Departments of Hepatology, Graduate School of Medicine, Osaka Metropolitan University, 1-4-3, Asahi-Machi, Abeno-Ku, Osaka, Osaka 545-8585 Japan; 20 Department of Gastroenterology and Hepatology, Suita Municipal Hospital, 5-7, Kishibe Shinmachi, Suita, Osaka 564-8567 Japan; 21 Department of General Internal Medicine2, Kawasaki Medical School, Kawasaki Medical Center, 2-6-1, Nakasange, Kita-Ku, Okayama, Okayama 700-8505 Japan; 22 Department of Gastroenterology, JA Hiroshima Kouseiren General Hospital, 1-3-3, Jigozen, Hatsukaichi, Hiroshima 738-8503 Japan; 23 Hyogo Life Care Clinic Hiroshima, 6-34-1, Enkobashi-Cho, Minami-Ku, Hiroshima, Hiroshima 732-0823 Japan; 24 Department of Gastroenterology and Neurology, Faculty of Medicine, Kagawa University, 1750-1, Oaza Ikenobe, Miki-Cho, Kita-Gun, Kagawa 761-0793 Japan; 25 Department of Gastroenterology and Hepatology, Gifu Municipal Hospital, 7-1, Kashima-Cho, Gifu, Gifu 500-8513 Japan; 26 Division of Hepatology, Shimane University Hospital, 89-1, Enya-Cho, Izumo, Shimane 693-8501 Japan; 27 Division of Gastroenterology and Hepatology, Department of Internal Medicine, National Defense Medical College, 3-2, Namiki, Tokorozawa, Saitama 359-8513 Japan; 28 Division of Gastroenterology and Hepatology, Tokyo Medical University Ibaraki Medical Center, 3-20-1, Chuo, Ami-Machi, Inashiki-Gun, Ibaraki, 300-0395 Japan; 29 Liver Center, Faculty of Medicine, Saga University Hospital, Saga University, 5-1-1, Nabeshima, Saga, Saga 849-8501 Japan; 30 Department of Gastroenterology and Hepatology, Yokohama City University School of Medicine Graduate School of Medicine, 3-9, Fukuura, Kanazawa-Ku, Yokohama, Kanagawa 236-0004 Japan; 31 Department of Internal Medicine, Hanyang University College of Medicine, Seoul, 04763 Korea; 32 Division of Hepatology and Pancreatology, Department of Internal Medicine, Aichi Medical University, 21 Yazako Karimata, Nagakute, Aichi 480-1195 Japan; 33 Department of Gastroenterology & Hepatology, Saiseikai Suita Hospital, Osaka, 1-2, Kawazono-Cho, Suita, Osaka 564-0013 Japan

**Keywords:** NAFLD/NASH, Scoring system, Biomarker, Elastography, Artificial intelligence

## Abstract

Nonalcoholic fatty liver disease (NAFLD) is the most common chronic liver disease. Nonalcoholic steatohepatitis (NASH) is an advanced form of NAFLD can progress to liver cirrhosis and hepatocellular carcinoma (HCC). Recently, the prognosis of NAFLD/NASH has been reported to be dependent on liver fibrosis degree. Liver biopsy remains the gold standard, but it has several issues that must be addressed, including its invasiveness, cost, and inter-observer diagnosis variability. To solve these issues, a variety of noninvasive tests (NITs) have been in development for the assessment of NAFLD progression, including blood biomarkers and imaging methods, although the use of NITs varies around the world. The aim of the Japan NASH NIT (JANIT) Forum organized in 2020 is to advance the development of various NITs to assess disease severity and/or response to treatment in NAFLD patients from a scientific perspective through multi-stakeholder dialogue with open innovation, including clinicians with expertise in NAFLD/NASH, companies that develop medical devices and biomarkers, and professionals in the pharmaceutical industry. In addition to conventional NITs, artificial intelligence will soon be deployed in many areas of the NAFLD landscape. To discuss the characteristics of each NIT, we conducted a SWOT (strengths, weaknesses, opportunities, and threats) analysis in this study with the 36 JANIT Forum members (16 physicians and 20 company representatives). Based on this SWOT analysis, the JANIT Forum identified currently available NITs able to accurately select NAFLD patients at high risk of NASH for HCC surveillance/therapeutic intervention and evaluate the effectiveness of therapeutic interventions.

## Introduction

Nonalcoholic fatty liver disease (NAFLD) is the most common liver disease worldwide, and its progression to nonalcoholic steatohepatitis (NASH) and fibrosis contribute to a growing proportion of the population with cirrhosis and hepatocellular carcinoma (HCC) [1]. Currently, liver biopsy remains the gold standard for diagnosis of NAFLD/NASH, although it has several issues that must be addressed, such as its invasiveness [2] and cost, sampling errors [3], and inter-observer variability. Hepatic fibrosis evaluated by liver histology is independently associated with overall mortality or liver-related events in the US, Europe, and Japan [4, 5]. Therefore, noninvasive tests (NITs) should be required to identify the disease severity of NAFLD.

The Japanese Society of Gastroenterology and the Japanese Society of Hepatology established the Japanese NAFLD/NASH guidelines in 2014 [6, 7] and revised these guidelines in 2020 [8, 9]. The guidelines have received considerable attention and have been widely used in clinical applications, including NITs. They recommend the fibrosis-4 index (FIB-4) and/or the NAFLD fibrosis score (NFS) for classifying high-risk NAFLD patients. As a first step, family physicians or general practitioners at medical check-ups examine liver fibrosis–related markers (FIB-4, NFS, platelet count, enhanced liver fibrosis [ELF] test, Mac-2 binding protein glycosylation isomer [M2BPGi], autotaxin [ATX], type 4 collagen 7S [T4C7S], hyaluronic acid [HA], etc*.*) in patients with fatty liver for the primary screening. A neo-epitope pro-peptide of type III collagen formation (PRO-C3) is also a useful liver fibrosis biomarker. An algorithm incorporating PRO-C3 has been reported to better identify patients with NAFLD and advanced fibrosis than either the NFS or FIB-4 index alone [10]. However, PRO-C3 testing is not currently covered by insurance in Japan, and insurance coverage is long awaited. Patients with a low risk of advanced hepatic fibrosis (FIB-4 < 1.3 or NFS <  − 1.455) do not need further assessment. If a patient is diagnosed with possible advanced hepatic fibrosis (FIB-4 ≥ 1.3, NFS ≥  − 1.455, or platelet count < 200,000/mm^3^), general practitioners should consult with a hepatologist, who conducts the second step. Hepatologists first check FIB-4 or NFS. If intermediate risk for liver fibrosis (FIB-4 1.3–2.66 or NFS − 1.455–0.674) or severe liver fibrosis (FIB-4 ≥ 2.67 or NFS ≥ 0.675) are suspected, liver biopsy or elastography (ultrasonography, magnetic resonance imaging [MRI]) is recommended. Surveillance for HCC and cardiovascular disease (CVD) are also recommended for patients with possible advanced hepatic fibrosis.

Various NITs are available for NAFLD, including biomarkers and imaging tests, but each NIT has strengths and weaknesses. It is necessary to sort out the strengths and weaknesses of the NITs and combine them or create novel NITs. To achieve this goal, we held a SWOT (strengths, weaknesses, opportunities, and threats) analysis discussion at the Japan NASH NIT (JANIT) Forum, which was organized in 2020. A SWOT analysis is a useful strategy for optimizing resource management in response to changes in the business environment by analyzing external and internal environments in four categories and projecting which organizations and individuals need to make decisions to achieve specific goals. Recently, this method has also been used in the field of gastroenterology [11]. The JANIT Forum aims to advance the development of various NITs to diagnose and assess the response to treatment for NAFLD from a scientific perspective through multi-stakeholder dialogue with open innovation including clinicians with expertise in NAFLD, companies developing medical devices and biomarkers, and professionals in the pharmaceutical industry.

In JANIT Forum, each member was a professional from a healthcare-related company or administrative organization or a healthcare professional involved in the treatment of NAFLD/NASH, who agreed to the purpose of the JANIT Forum and committed themself to discussing the information obtained at the JANIT Forum from a scientific point of view without giving priority to the interests of their own organization. This SWOT analysis discussion was a joint initiative of physicians and professionals from medical device companies, pharmaceutical companies, and diagnostics companies. Although the SWOT framework is most commonly employed in business to analyze the factors that influence a company’s position in the marketplace with a focus on the future, it can also be useful for other domains, such as in the scientific field [12]. The SWOT analysis discussion had 36 participants: 16 physicians from 15 hepatology centers and 20 company representatives from 10 companies. The method of analyzing SWOT was not restricted but freely discussed; it included initial individual SWOT analyses, bringing the results to the group, setting subgroups for various diagnostic methods and creating SWOT in each team, cross-SWOT analysis, selecting key success factors from cross-SWOT analysis, and prioritizing. Our discussion took place primarily online using tools such as Zoom, Microsoft Teams, Facebook, and Slack due to the COVID-19 situation.

## Strengths and weaknesses of each NIT

We first presented the strengths and weaknesses of each NIT used in Japan (Table 1). The approval status and price of each NIT are demonstrated in Table 2.

1. **Simple index (scoring system)**
2. **FIB-4 index**
  The strengths of the FIB-4 are its simplicity, accuracy, and validation: (i) FIB-4 is based only on the combination of four parameters—age, aspartate aminotransferase (AST), alanine aminotransferase (ALT), and platelets—which are measured as part of the liver blood test [13, 14]. FIB-4 can be easily calculated and is widely available in clinical settings at a low cost. (ii) The diagnostic accuracy of FIB-4 for advanced fibrosis is superior to that of other blood-based NITs, such as NFS, AST to platelet ratio index (APRI), and the body mass index (BMI), AST/ALT ratio, and diabetes (BARD) score [15–20]. In addition, FIB-4 can act as a predictor of incident HCC [21–24], CVD [25, 26], liver-related events [27–29], and mortality. (iii) FIB-4 is the most validated in the prediction of NAFLD with severe liver fibrosis, and some clinical practice guidelines have recommended it as a first triaging tool in clinical practice [8, 30, 31].
  A weakness of FIB-4 is that its sensitivity to predict advanced fibrosis is lower in certain populations: (i) Age affects the accuracy of FIB-4, which might lead to overpredicting fibrosis in older adults (> 65 years) [32, 33]. (ii) FIB-4 has shown lower performance in predicting advanced fibrosis in obese NAFLD patients than in non-obese patients [34]. (iii) FIB-4 may less accurately predict fibrosis in NAFLD patients with type 2 diabetes mellitus (T2D) compared to those without T2D [35, 36]. (iv) As FIB-4 was validated in populations with a high prevalence of chronic liver diseases, lower positive predictive values have been reported in low-prevalence populations, such as the general population [30, 37].
3. **NFS**
  NFS is a validated, noninvasive tool for identifying patients whose NAFLD has advanced to liver fibrosis and is based on six available variables: age, BMI, hyperglycemia, platelet count, albumin, and the AST/ALT ratio [38]. A published formula is also available at https://nafldscore.com/.
  Its strengths are as follows: i) The NFS variables consist of routine clinical and laboratory data [38]. ii) The diagnostic accuracy of NFS is almost the same as that of FIB-4 [39]. iii) NFS is listed and recommended as a scoring system to screen for advanced liver fibrosis/HCC, alongside FIB-4, in the clinical practice guidelines of Japan [8, 9]. iv) NFS is recommended by the clinical practice guidelines of both the European Association for the Study of the Liver and American Association for the Study of Liver Diseases [30, 31]. (v) NFS is useful in identifying NAFLD/NASH patients with T2D at low or high risk for advanced fibrosis [31]. (vi) NFS is one of the most popular noninvasive blood-based serum tests; therefore, a huge amount of data has been published.
  In contrast, the weaknesses of NFS are as follows: (i) Obesity affects the performance of NFS [40, 41]. (ii) The diagnostic accuracy of NFS is not very high compared with other NITs [42]. iii) NFS has reduced specificity in elder patients [32]. (iv) In T2D patients, the NFS tends to be high and difficult to use for exclusion diagnosis [43]. (v) The NFS formula is complicated [38].
4. **Hepamet fibrosis scoring (HFS)**
  Recently, the Hepamet fibrosis scoring (HFS) system was developed based on clinical and laboratory test results, such as age, sex, levels of AST and albumin, homeostatic model assessment score (HOMA), presence of diabetes mellitus, and platelet count. HFS shows greater accuracy than the FIB-4 and NFS scoring systems among European NAFLD patients with advanced fibrosis [17][17]. By contrast, HFS has been reported to have lower diagnostic efficacy for F3–4 than FIB-4 among patients with biopsy-confirmed NAFLD from Asia [45].
5. **Liver-specific fibrosis markers**
6. **ELF test**
  The ELF test is a scoring system that diagnoses liver fibrosis and gives prognostic insight into the occurrence of liver-related events. It is calculated from serum values of HA, type III procollagen-N-peptide, and tissue inhibitor of metalloproteinase 1. As with other serum markers, the advantages of the ELF test are that it is minimally invasive and can be performed easily and repeatedly and without the installation of special equipment. The National Institute for Health and Care Excellence guidelines recommend the ELF test for identifying advanced liver fibrosis patients [46]. In Europe, the ELF test has been proposed as one of the patented serum fibrosis markers to be measured after FIB-4 and transient elastography (FibroScan) in the patient selection algorithm [30]. Measuring FIB-4 followed by the ELF test has resulted in an 85% reduction in unnecessary referrals compared with when FIB-4 and ELF test were not used [47]. In Japan, the ELF test has not yet been approved for clinical settings; thus, evidence regarding Japanese patients is limited. However, high diagnostic performance was reported as the areas under the curve (AUCs) of NAFLD fibrosis stages ≥ F2 and ≥ F3 were 0.826 and 0.812, respectively, and the diagnostic accuracy of the ELF test is comparable to that of FibroScan [48].
7. **T4C7S**
  T4C7S is a major component of the lamina densa of the basement membrane. The basement membrane is formed with liver fibrillation, causing the T4C7S concentration in the blood to rise. It is higher in chronic hepatitis and liver cirrhosis patients than in acute hepatitis patients, especially in cases with high inflammatory activity. T4C7S has been used since 1989 and was introduced in the Japanese NAFLD/NASH guidelines in 2014 [6, 7]. The T4C7S assay method has historically been radio immunoassay (RIA) [49], although it recently changed to chemiluminescent enzyme immunoassay (CLEIA). The sensitivity and specificity of CLEIA for the detection of the fibrosis degree of NAFLD patients have been improved compared with RIA [50]. The AUC of T4C7S (CLEIA) for diagnosing liver fibrosis stages ≥ 2 in NAFLD patients was 0.882 and that of RIA was 0.855, making it an important fibrosis marker for early fibrosis. Studies of NAFLD patients with or without T2D, especially in the NAFLD group with T2D, have reported that this marker is superior to other hepatic fibrosis markers [35]. In a report from Shinshu University, the AUCs for fibrosis stages ≥ 3 were 0.87 for all subjects, 0.81 for men, and 0.89 for women [51]. In a report at Yokohama City University, the AUC for fibrosis stages ≥ 2 was 0.83 for both men and women [52]. Insurance fees in Japan for T4C7S are low among other noninvasive markers, including other fibrosis markers and imaging, and can contribute to the healthcare economy. Although T4C7S is widely used in Japan, it is not used in other countries, so its future utilization is expected to grow.
8. **M2BPGi**
  Mac-2 (galectin-3) binding protein (M2BP) is a glycoprotein that has seven potential *N*-glycosylation sites [53, 54]. M2BP is barely detectable in a normal liver but is strongly detected in hepatocytes from chronic hepatitis type C (CHC) patients as liver fibrosis progresses [55, 56]. In addition, the structure of M2BP glycans has been reported to be markedly altered by fibrosis progression in the liver [57]. M2BPGi is a serum liver fibrosis biomarker and a glycosylation isomer that is recognized by *Wisteria floribunda* lectin (also known as WFA[ +]-M2BP) [57]. This marker is a useful predictor of NAFLD at fibrosis stages ≥ 2 and ≥ 3 [58], is not affected by age, and can be judged by a single cutoff point [59]. In addition, M2BPGi can differentiate patients at high risk for severe fibrosis from a healthy control group [60], and it may be a predictor of hepatocarcinogenesis, though further studies are required [61]. The M2BPGi clinical test is reimbursable in Japan, but limited data are available in other Asian-Pacific countries as highlighted in the 2016 Asian Pacific Association for the Study of the Liver consensus guidelines. [62]
  Because M2BPGi was identified and developed as a fibrosis marker from the serum of patients with CHC [57], its behavior differs based on the level of fibrosis progression against the background of other etiologies. Therefore, cutoffs for different etiologies should be established [63]. Also, the pathophysiological mechanism of M2BPGi is unclear [61]. M2BPGi is a dedicated reagent for the HISCL system (Sysmex Co., Hyogo, Japan) and is currently registered only in Asia.
9. **ATX**
  ATX is a secreted enzyme that produces lysophosphatidate from extracellular lysophosphatidylcholine. Metabolized by liver sinusoidal endothelial cells, ATX is considered to be associated with liver damage. Serum ATX is a useful marker for diagnosing liver fibrosis in patients with NAFLD [51, 52]. As ATX levels are less affected by inflammation, they can be used to detect liver fibrosis at an early stage in Japanese patients with NAFLD [52]. Fujimori et al*.* reported an AUC of 0.75 for the efficacy of serum ATX in diagnosing liver fibrosis stages ≥ 3 for all patients with NAFLD (AUC 0.74 for male patients and 0.78 for female patients) [51]. Honda et al*.* also reported AUCs of 0.75 and 0.81 for the efficacy of serum ATX in diagnosing liver fibrosis stages ≥ 2 in male and female NAFLD patients, respectively [52]. However, it should be noted that the reference values of ATX are different between men and women, higher values are observed in pregnant women and patients with follicular lymphoma, and ATX levels are not generally measured in Europe or the US for diagnostic purposes.
10. **HA**
  HA is an acidic mucopolysaccharide obtained by polymerizing D-glucuronic acid and *N*-acetyl-D-glucosamine, which are mainly produced in fibroblasts and synovial cells. Due to the decrease in HA receptors with liver fibrosis progression, serum HA levels become high. HA has long been known to be a useful marker of liver fibrosis. Loomba et al*.* reported an AUC of 0.812 for the efficacy of serum HA in differentiating between liver fibrosis stages 0–2 and 3–4 in patients with NAFLD in the US [64]. Fujimori et al*.* reported an AUC of 0.82 for the efficacy of serum HA in diagnosing liver fibrosis stages ≥ 3 for Japanese patients with NAFLD [51]. It has also been reported that the combination of FIB-4 and serum HA is a better marker than FIB-4 alone with respect to predicting the occurrence of cirrhosis and HCC in patients with diabetes [65]. Furthermore, serum HA levels can be used to predict hepatic fibrosis in pediatric patients with NAFLD [66]. However, it should be noted that serum HA levels are elevated in patients with renal dysfunction, joint disorders (*e.g*., rheumatoid arthritis, osteoarthritis), scleroderma, dermatomyositis, vasculitis, and malignant cancers (*e.g.*, malignant lymphoma, breast cancer).
11. **Cytokeratin-18 fragment**
  Active caspases in NASH specimens have been reported to be strongly correlated with hepatocyte apoptosis and NASH progression [67]. Hepatocyte ballooning, a form of hepatocyte apoptosis, is a prominent pathological feature of NASH and an important component of the NAFLD activity score (NAS). Cytokeratin 18 (CK18), the major intermediate filament protein in the liver, is cleaved by caspases during hepatocyte apoptosis. Cleaved CK18 shed into the blood has been reported as a biomarker of hepatocyte apoptosis [68]. Feldstein et al*.* first demonstrated that circulating levels of CK18 fragment (CK18-F/M30) were a predictor of NASH in NAFLD patients [69]. Since then, the usefulness of this marker in differentiating between simple steatosis and NASH has been demonstrated in several clinical studies [70, 71].
  However, subsequent studies have revealed its limited sensitivity at the individual level and concluded that it is inadequate as a screening test for diagnosis and staging of NASH [72, 73]. Even still, the involvement of CK18 in the specific disease pathway of NASH suggests the potential for CK18-F to be used in combination with other NITs. Recent studies have shown that the accuracy of NASH diagnosis is improved by the combination of CK18-F with other biomarkers (*e.g.*, hyaluronic acid) or scoring systems (e.g., FIB-4) [74–76]. It is noteworthy that we can more easily diagnose NASH in combination with CK18-F even in cases with a low FIB-4, NFS score, or TE value [75–77], suggesting its ability to rule in NASH among patients rated at low or intermediate risk by clinically established NITs.
  The CK18-M30 enzyme-linked immunosorbent assay (ELISA) kit, commercially available for research use only, has not been readily translatable to clinical settings due to variations in disease marker cutoff values and diagnostic performance issues [31]. Based on data showing that adding CK18-F to fibrosis markers can be useful for screening NAFLD patients for NASH, an ELISA kit for CK18-F measurement was finally approved as an in vitro diagnostic reagent in Japan in 2021. It is expected that the significance of this marker will become clear as this reagent becomes widely used in clinical settings in the future.
12. **Elastography**
  As of recently, we can use various liver stiffness measurement (LSM) methods that are about to replace liver biopsy. Vibration-controlled transient elastography (VCTE, or FibroScan), point shear wave elastography (p-SWE), 2-dimensional SWE (2D-SWE), and magnetic resonance elastography (MRE) are available in Japan. We summarized the respective characteristics, advantages, and limitations of the four available elastography techniques for liver fibrosis staging (Table 3). In addition, applications are being developed by each manufacturer as a quantitative evaluation method for hepatic steatosis. They have been newly reimbursed since 2022. There are methods for measuring the attenuation coefficient—*e.g.*, the controlled attenuation parameter (CAP; Echosens), ultrasound-derived fat fraction (Siemens Healthcare), attenuation imaging (Canon Medical Systems), attenuation coefficient measurement (Fujifilm), and ultrasound-guided attenuation parameter (GE Healthcare)—and some vendors add the backscatter coefficient to measure it.
13. **VCTE**
  VCTE (FibroScan) is an NIT that has been widely validated around the world since it was launched in Europe in 2003. In Japan, it was launched and reimbursed in October 2011. On April 16, 2013, Echosens announced that its FibroScan device received 510(k) clearance from the US Food and Drug Administration (FDA). For NAFLD, the utility of LSM by VCTE to assess liver fibrosis was first validated in Japan in 2008 by Yoneda et al. [78]. A recent meta-analysis [79] (VCTE: 53 papers) reported that VCTE has excellent diagnostic performance with an AUC of 0.82 for fibrosis stages ≥ 1, 0.83 for stages ≥ 2, 0.85 for stages ≥ 3, and 0.89 for stage 4. As a quantitative steatosis assessment method, CAP was developed and installed into FibroScan to measure ultrasound attenuation and has been globally validated since 2010 [80, 81].
  VCTE is a safe and simple method that also can be used with pregnant patients [82, 83]. The use of VCTE is limited in patients with ascites and narrow intercostals [84, 85]. For obese patients, VCTE can be conducted using an XL probe, but this is difficult in patients with severe obesity [86, 87]. SmartExam, which has recently launched, is expected to extend VCTE usage among severely obese patients [88] and improve the reliability and precision of CAP with reduced variability by the continuous CAP method [89].
  Reported confounding factors for LSM by VCTE to assess fibrosis include not only obesity [86, 87, 90–92] but also inflammation [92], food intake s, biliary obstruction [93], heart failure [94], amyloidosis [95], solitary liver lesions [96], and portal hypertension (PH) [97]. Elevated LSM by PH is significantly correlated with the hepatic venous pressure gradient in patients with advanced chronic liver disease/compensated cirrhosis and has been applied to predict the presence of esophageal varices [97]. Spleen stiffness measurement (SSM) by VCTE is reported to be more accurate for prediction than LSM by VCTE [98] and a more specific model for SSM (FibroScan630Expert) has recently been developed [99]. Operator experience might influence the diagnostic performance of VCTE as well [100].
  FibroScan-AST (FAST) score which combined LSM by VCTE, CAP for a quantitative steatosis assessment method, and AST increases the diagnostic accuracy to identify active fibrotic NASH patients which is defined NASH with significant fibrosis (stages ≥ 2) and NAS ≥ 4 [101, 102]. In pharmaceutical trials for NASH drug pipelines, LSM and CAP have been referred to as alternative methods for liver biopsy [103], and LSM by VCTE, CAP and FAST score has been adopted in many trials [104–108].
14. **p-SWE/2D-SWE**
  Ultrasound SWE uses acoustic radiation force impulses (ARFI) or mechanical impulse to stimulate liver tissue to produce shear waves that propagate through the liver. The shear wave velocity (SWV) increases with the severity of fibrosis. The ARFI method uses both p-SWE, which measures the region of interest (ROI) by setting one point [109], and 2D-SWE, which measures the SWV by color mapping [110]. In other words, p-SWE generates displacement at a single focal point, whereas 2D-SWE is a dynamic displacement method that can generate stress in multiple focal zones with the same ARFI technique. In Japan, p-SWE and 2D-SWE are approved for the examination of patients with cirrhosis or suspected cirrhosis and reimbursed in October 2016. Both p-SWE and 2D-SWE can be performed at the same time as ultrasound imaging, which is an advantage in that it can be easily introduced at a facility. In the mechanical impulse method, VCTE is recommended in Europe to exclude and diagnose compensated advanced chronic liver disease, which is defined as fibrosis stages ≥ 3 [111]. p-SWE and 2D-SWE may perform similarly to VCTE, and direct comparisons of p-SWE and 2D-SWE with VCTE have been reported [112]. Similar to VCTE, p-SWE and 2D-SWE have been reported to be useful for evaluating hepatic fibrosis in NAFLD [113, 114]. In addition, 2D-SWE and MRE have demonstrated excellent accuracy in diagnosing liver fibrosis in NAFLD [114] and alcoholic liver disease [113]. Furthermore, 2D-SWE has been used in conjunction with the FIB-4 index to assess hepatic fibrosis in NAFLD, metabolic-associated fatty liver disease (MAFLD), and health checkup examinees [113, 115, 116]. The measurement value of chronic liver disease is different by manufacturer and model, so attention to this is necessary [30]. Confounders other than stiffness include non-fasting conditions, elevated aminotransferases, congestive heart failure, and extrahepatic cholestasis.
15. **MRE/proton density fat fraction**
  MRE is an MRI-based technique for the quantitative imaging of liver stiffness [117]. Liver stiffness maps can be obtained with one breath-hold and can be easily included in routine liver MRI protocols. MRE has been shown to be the most accurate imaging tool to assess liver fibrosis [118] in a geographically distinct cohort [119], even in the early stages [120] and in patients with ascites or obesity [121]. Because of this variety of evidence, the FDA approved MRE in 2009, and MRE has been newly reimbursed since 2022 in Japan. Optimal MRE thresholds for the detection of liver fibrosis stages are 2.61 kPa (stages ≥ 1), 2.97 kPa (stages ≥ 2), 3.62 kPa (stages ≥ 3), and 4.69 kPa (stages ≥ 4) [122]. Moreover, MRE can visualize whole-liver stiffness, resulting in reduced sampling error [123], and be readily combined with other quantitative maps, such as proton density fat fraction (PDFF) and R2* [124]. However, MRE also has weaknesses—it is inaccessible, costly, and time-consuming compared with ultrasound methods. Inter-observer bias in ROI placement may be one of the most critical issues for MRE quantification, but an automatic ROI-drawing tool using artificial intelligence (AI) [125] is expected in the near future.
  PDFF is also an important MRI-based biomarker to quantitatively measure hepatic fat accumulation, which correlates with the histologically determined steatosis grade [126]. It exploits the chemical shift–encoded MRI method to accurately quantify the relative amount of water and fat signal and calculates the ratio of the density of protons from triglycerides and the total density of protons from both mobile triglycerides and water [127]. PDFF is expressed as an absolute percentage (%), and its thresholds for the detection of liver steatosis grades are: 5.2% (grades ≥ 1), 11.3% (grades ≥ 2), and 17.1% (grades ≥ 3) [118]. Combining MRE with PDFF has been shown to improve the diagnosis of NASH [128], and the accuracy of these MRI-based imaging biomarkers can contribute to evaluating the efficacy of clinical trials [129].
16. **AI**
17. **Background**

**Table 1:** Strengths and Weaknesses of NITs for NAFLD available in Japan

		Strengths	Weaknesses
Scoring system	FIB-4	Easy calculation with low cost High negative predictive value for advanced fibrosis Possible predictor of incident HCC, CVD, and mortality Widely validated score recommended as first triaging tool for clinical practice Rich evidence	Low performance in older patients (> 65 years) Lower performance for advanced fibrosis in obese NAFLD patients than for nonobese NAFLD patients Unlikely to be accurate for fibrosis in NAFLD patients with T2D compared with those without T2D Lower positive predictive values in low-prevalence populations, such as the general population Existence of indeterminate group
	NFS	Easy utilization with clinical and laboratory data Recommended in the clinical practice guidelines of both EASL and AASLD Identification of NAFLD/NASH patients with T2D at low or high risk of advanced fibrosis Rich evidence	Low performance in obese patients Lower diagnostic accuracy than other NITs Low performance in older patients Complex formula Existence of indeterminate group
	HFS	Easy utilization with clinical and laboratory data Possible predictor of mortality in European populations	Less evidence Lower diagnostic efficacy than FIB-4 index in Asian populations Not well known in Japan
Biomarker	T4C7S	Well measured in Japan Reflect fibrosis formation in the liver Low insurance fee	Not generally measured outside of Japan due to its low awareness
	ATX	Less affected by inflammation Can be used to detect liver fibrosis at an early stage in Japanese patients with NAFLD	Difference between men and women Not generally measured outside of Japan
	HA	Well known to be a useful marker of liver fibrosis Useful in pediatric patients with NAFLD	Elevation of serum HA level in patients with renal dysfunction, joint disorders, and malignant cancers
	CK-18F (M30)	Predictor of hepatocyte apoptosis Recently approved as NASH diagnosis marker in Japan	Less evidence Cutoff values not established for the diagnosis of NASH
	M2BPGi	Better predictor of fibrosis stages ≥ 2 and ≥ 3 Single cutoff value independent of age High performance of differentiating high-risk patients with advanced fibrosis from the general population Possible predictor of hepatocarcinogenesis	Different cutoffs for different etiologies Unclear mechanism of action Dedicated equipment required (HISCL Series system by Sysmex Co.) Not a quantitative assay
	ELF test	Easy to use (repeatable, minimally invasive, no equipment installation or special training required) Recommended in Europe as a patented fibrosis marker Approved by the FDA as a NASH prognosis marker (used to identify high-risk NASH patients)	Not approved for clinical use in Japan Limited evidence in Japanese patients Dedicated equipment is required for in-hospital diagnosis (Atellica or Centar series by Siemens Healthcare Diagnostics Co.)
Elastography	VCTE	Excellent diagnostic performance for the assessment of liver fibrosis Widely validated method around the world Easy to learn Quantitative assessment of steatosis using CAP Assessment of progressive NASH using FAST score	Limited in patients with ascites, narrow intercostal space, and severe obesity Confounders other than stiffness include nonfasting conditions, elevated aminotransferases, congestive heart failure, and extrahepatic cholestasis
	p-/2D-SWE	Approved for the examination of patients with cirrhosis or suspected cirrhosis in Japan Both p-SWE and 2D-SWE may be conducted concurrently with ultrasound imaging	Difference by manufacturer and model Factors other than stiffness include nonfasting conditions, elevated aminotransferases, congestive heart failure, and extrahepatic cholestasis Not an evaluation of the liver as a whole
	MRE	Best Accuracy for the assessment of liver fibrosis degree Good visibility of the whole liver Can be combined with fat (PDFF), corrected T1, and iron quantification (R2*) Can be applied to patients with ascites or obesity	(Compared to ultrasound) Inter-observer variability of ROI placement Inaccessibility Costly and time consuming Artifacts due to iron overload
Others	AI	Numerous analyses are possible using easily available information Reductions in cost, time, and human resources High accuracy	Black box nature Potential for leaks of private information Need for good training data

NIT, noninvasive test; NAFLD, nonalcoholic fatty liver disease; T2D, type 2 diabetes mellitus; EASL, European Association for the Study of the Liver; AASLD, American Association for the Study of Liver Diseases; FIB-4, Fibrosis-4; NFS, NAFLD fibrosis score; HFS, Hepamet fibrosis score; HCC, hepatocellular carcinoma; T4C7S, type 4 collagen 7S; ATX, autotaxin; HA, hyaluronic acid; CK-18F, cytokeratin-18 fragment; M2BPGi, Mac-2 binding protein glycosylation isomer; ELF, enhanced liver fibrosis; VCTE, vibration controlled transient elastography; CAP, controlled attenuation parameter; SWE, shear wave elastography; p-SWE, point SWE; 2D-SWE, 2-dimensional SWE; MRE, magnetic resonance elastography; PDFF, proton density fat fraction; AI, artificial intelligence; ROI, region of interest; T2D, type 2 diabetes

**Table 2:** Approval status in USA, EU, and Japan, and price of each NIT in Japan

NITs		Registration/Approval			Status in Japan	
		USA	EU	Japan	Reimbursement	Fee (JPY)
Scoring system	FIB-4	N/A	N/A	N/A	N/A	N/A
	NFS	N/A	N/A	N/A	N/A	N/A
Liver-specific fibrosis markers	ELF test	○	○	-	-	-
	T4C7S	-	-	○	○	1,480
	M2BPGi	-	-	○	○	1,940
	ATX	-	-	○	○	1,940
	HA	-	○	○	○	1,790
Apoptosis marker	CK-18F (M30)	-	-	○	-	-
Liver stiffness measurement	VCTE	○	○*	○	○	2,000
	p-/2D-SWE	○	○	○	○	2,000
	MRE	○	○	○	○	6,000
Liver fat measurement	Attenuation Coefficient (CAP, etc.)	○	○*	○	○	2,000
	PDFF	○	○	○	-	-

(As of April 2022)

Annotation. ○ indicates “Available,”—indicates “Not available.”

Registration/Approval means FDA approval in the USA, CE marking in the EU, and Ministry of Health, Labour and Welfare or Registered Certification Bodies approval in Japan

^*^FibroScan, which measures LSM by VCTE and CAP, is a CE-marked class IIa ultrasound diagnostic medical device (hepatic and/or splenic applications)

**Table 3:** Respective characteristics, advantages, and limitations of the 4 available elastography techniques for liver fibrosis staging

Imaging	Range (units)		Steatosis grading	Quality criteria	Confounders		
					Inflammation	Obesity	Others
VCTE	1.5 ~ 75.0	kPa	Yes (CAP)	IQR/M ≤ 30%	+	+	Food intake Biliary obstruction Heart failure Amyloidosis Solitary liver lesions Portal hypertension Operator experience
p-/2D-SWE	0.5 ~ 6.5	m/s	Yes (UDFF, ATI, ATT, UGAP)	IQR/M ≤ 15%	+	+	Food intake Obstructive cholestasis Liver congestion Acute hepatitis Infiltrative liver disease
MRE	0 ~ 20	kPa	Yes (PDFF)	Based on QIBA consensus statement	+	+	Hemochromatosis/ Hemosiderosis Claustrophobia Metal implant

QIBA, Quantitative Imaging Biomarkers Alliance; UDFF, ultrasound-derived fat fraction (Siemens Healthcare); ATI, attenuation imaging (Canon Medical Systems); ATT, attenuation measurement method (Fujifilm); UGAP, ultrasound-guided attenuation parameter (GE Healthcare)

**Table 4:** Opportunities and Threats of NITs for NAFLD

PEST	Opportunities (O)	Threats (T)
Politics	The MHLW has attempted to increase the rate of acceptance of Specific Health Checkups and promote regional coordination of local clinics and hospitals There is a need for NITs that can be easily used by primary care doctors to correctly identify people with advanced liver fibrosis	Authorities might consider NITs insufficient as a complete substitute for liver biopsy due to the lack of evidence Platelet count is not available for health checkup examinees covered by National Health Insurance, meaning some scores of NITs are not calculated
Economics	The MHLW attempts to reduce total health expenditures to maintain the universal insurance system The development of an inexpensive NIT using blood samples for correct prognosis of NAFLD is expected	Current NITs may not be used any longer if cheap and easy-to-use NITs are developed in the future Facilities equipped with elastography are limited, especially in rural areas with few patients and limited budgets Overall medical costs will soar with frequent use of expensive NITs for NAFLD screening
Society	The number of new subscribers for health apps is increasing Simple and highly accurate NITs are required for non-specialist and primary care doctors	Physicians are less motivated to use NITs for reasons that include the lack of therapeutic agents for NAFLD Insufficient cooperation between hepatologists, diabetologists, cardiologists, and primary care clinicians will make the use of NITs challenging Physicians cannot use NITs measured at other institutes for same-day diagnosis If different NITs are established by region or country, it will be difficult to find a consensus between regions
Technology	With the spread of 5G networks, advanced imaging technology and online medical care are more accessible Doctors and patients need NITs that allow simple visualization and easily understanding of NAFLD status	Physicians may stop using current NITs if novel NITs with higher diagnostic performance or NITs without blood sampling (*e.g.*, wearable devices) are developed in the future

MHLW, Ministry of Health, Labour and Welfare; NIT, noninvasive therapy; NAFLD, nonalcoholic fatty liver disease

AI is going to be deployed in many areas of the NAFLD landscape [130]. The origin of AI for healthcare was developed in 1954 [131], and there have been several booms and chasms since then [132]. Information and communication technology has been making drastic changes since 2000 [133]. Although AI needs big data and faster computers, the past weaknesses and limitations were resolved by an advance in the environment around AI models [134]. Currently, the need for AI in NAFLD-related diagnostics is expanding.

(2) **Strengths of AI in the NAFLD/NASH area**
  The overall strengths of AI include (i) the possibility of performing numerous analyses using easily available information; (ii) reductions in cost, time, and human resource needs; and (iii) high accuracy.
  Although NITs are expected to identify patients with advanced NAFLD, AI can expand the possible analyses [135], such as comparing healthy patients with patients diagnosed with NAFLD subjects or comparing NAFLD patients with comorbidities to those without comorbidities. Thus, AI has the potential to both identify NAFLD cases and assess NASH severity, including comorbidities such as HCC or cardiovascular disease.
  Various information can be used to obtain an “AI diagnosis,” including the electronic health record (EHR), laboratory data, and imaging examinations. However, assessments of these data have been largely researcher-dependent. In addition, processing large amounts of data can cause physicians to be overworked, leading to human error [136, 137]. By contrast, AI enables us to make highly reproducible diagnoses without heavy workloads, leading to low intra- and inter-rater variability. The EHR is rich in information for the diagnosis of NAFLD. Fialoke et al*.* and Docherty et al*.* developed AI models isolating clinically meaningful values from the EHR under HIPAA compliance [138, 139]. A combination of AI and EHR data has been used not only for the diagnosis of NASH but also for the assessment of drugs used for NASH treatment. In addition, many AI models use clinical parameters, including physical examinations and laboratory data [140–142]. In general, radiological diagnosis for NASH entails heterogenous image reconstruction, segmentation, and quantification. In addition, shape, texture, volume, diffusion, and other parameters must be processed. AI automatically processes a large amount of digital data and increases the accuracy of diagnosis. Mojtahed et al*.* showed that Hepatica (Perspectrum, UK), a deep-learning system, could shorten the time required to assess the detailed hepatic volume and hepatic condition while maintaining high reproducibility compared with a conventional method. Conventional ultrasonography is a typical example of observer-dependent examination. AI can automatically classify ultrasound images [143] and SWE images [144] to reduce manual workload.
  Although early-stage “AI diagnosis” was not always accurate, current AI models provide amazing results. Zamanian et al*.* reported that the AUC for AI-equipped ultrasonography was 0.9999 for diagnosing NAFLD [145]. Okanoue et al*.* developed AI models using physical examinations and common laboratory data [146, 147]. The AUC was 0.995 when AI was applied to discriminate NAFLD from non-NAFLD. In addition, the AUC was 0.960 for the discrimination between NASH with and without fibrosis. Furthermore, the AI model can discriminate fibrosis staging with high accuracy.
(3) **Weakness of AI in NAFLD/NASH areas**

AI has some weaknesses, including (i) its black-box nature, (ii) the potential for leaks of private information, and (iii) the need for good teachers. First, it is difficult to know the decision-making process of an AI algorithm, which is an eternal weakness of AI. Second, it is crucial to protect privacy because private information in healthcare systems is sensitive and confidential. Although AI and the digital data of patients are inseparable, the FDA ensures that federal standards are maintained when the EHR is used for AI analyses [148]. Compliance with regulations can be the biggest barrier for regulatory approval. In addition, privacy should be protected from outsiders. Several Japanese hospitals have been attacked by hackers, resulting in potential breaches of data. To better safeguard these files, the Cyber Security Framework was issued by the National Institute of Standards and Technology in 2014. In addition, the Cyber Risk Intelligence Cross-Sector Forum was founded to execute cybersecurity in Japan. These systems now collaborate with each other and function globally to reduce weaknesses in data privacy. When information for AI analyses is restricted, privacy issues are reduced. Third, AI needs good-quality test data. Most test data have been based on liver biopsy, which has sampling variability [149] and other limitations [150]. The histologic scoring systems are semiquantitative with marked inter- and intra-observer variation. Thus, in this case, experienced teachers are not always good teachers. We should grow good teachers by using digital pathology and other clinical parameters, including imaging examinations.

## Opportunities and threats

Next, we analyzed the opportunities and threats in NITs based on the PEST (Politics, Economy, Society, Technology) perspective (Table 4) [151]. In Table 4, we discuss the opportunities and threats for all NITs.

1. **Opportunities for NITs**
  The Japanese Ministry of Health, Labour and Welfare (MHLW) has attempted to increase the rate of acceptance for Specific Health Checkups and promote regional coordination among local clinics and hospitals. The MHLW also attempts to reduce total health expenditures to maintain the universal insurance system. Therefore, the increasing development of NITs for NAFLD patients in Japan may be carefully considered. In addition, the number of new health app subscribers is increasing, which may result in increased NAFLD awareness, especially for the young to middle-aged population. Additionally, early diagnostic imaging for NAFLD is required to increase NAFLD awareness among non-specialist and primary care doctors. Furthermore, with the spread of 5G networks, advanced imaging technology and online medical care for NAFLD may be more accessible. Based on these opportunities for NITs, the following actions will be needed.
  To calculate the FIB-4 and NFS indexes for their primary screening described in the Japanese NAFLD/NASH guidelines [8, 9], attempts are required to enable non-specialist doctors to measure platelet counts and albumin for such calculations and enable hospitals and institutes to automatically calculate the indexes. Moreover, to correctly capture NAFLD and NASH status, we must ask MHLW to include measurements such as platelet count and albumin as diagnostic items based on the Industrial Safety and Health Act. In addition, new indexes calculated on the basis of measurements from current medical checkup items should be investigated.
  After patients diagnosed with liver fibrosis by screening are referred to a specialist, a simple imaging technique is required to provide clear diagnostic information. In particular, after new agents that provide indications for NAFLD are approved, a further simple imaging technique is expected such that non-specialist and primary care doctors can make diagnoses. Furthermore, making people aware of not only a liver disease itself but also the development of liver fibrosis is clinically important. Increasing awareness of imaging techniques that provide visually understandable information and health apps that allow users to check liver fibrosis progression from noninvasive indicators would be effective.
2. **Threats of NITs**

Currently, liver biopsy remains the gold standard for diagnosing NAFLD/NASH. In many clinical trials conducted on NASH patients, the primary outcome evaluation has been based on liver biopsy. MRE has been used instead of liver biopsy in some recent clinical trials, and the use of NITs is being considered for defining the trial population, assessing early treatment responses, and evaluating outcomes [152]. However, some NITs are expensive, and their frequent use will increase overall medical costs. These issues would make it difficult to use NITs for the assessment of NAFLD progression in patients.

To reduce the risk of death and poor prognosis due to NAFLD and reduce the burden on patients in the future, there is an urgent need to establish NITs that are highly diagnostic, inexpensive, easy-to-use, and compatible with global activities. To achieve this goal, we must understand the strengths and weaknesses of each NIT, develop combinations of NITs that complement each other, and accumulate evidence. Furthermore, a continuous educational campaign is needed so that patients have a high awareness of NASH and physicians understand the importance of identifying patients at high risk of NASH by using NITs.

## Future perspective (Fig. 1)

**Fig. 1:**
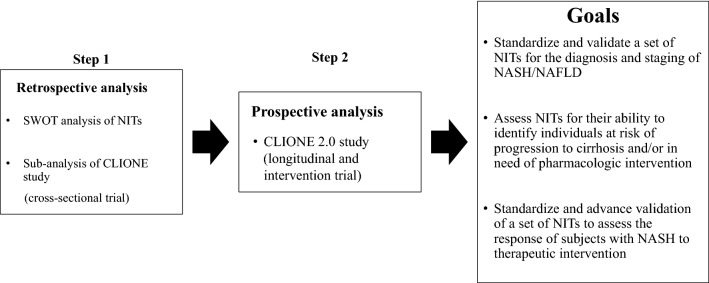
The JANIT Forum project plan Based on this SWOT analysis of NITs, a sub-analysis of the CLIONE study (cross-sectional trial in Japan) is underway. The next step of the JANIT Forum is the prospective CLIONE 2.0 study (longitudinal and intervention trial). Our goal is to establish standardized NITs for the assessment of NAFLD, which will enable us to diagnose disease severity and assess treatment response in NAFLD patients

Recently, our study group (Japan Study Group of NAFLD) disseminated data from the CLIONE study of a large cohort of Asian NAFLD patients [5]. We will perform sub-analyses of the CLIONE study to establish NITs in collaboration with companies in the JANIT Forum under a nondisclosure agreement for the next 3 years. We are currently planning the CLIONE 2.0 study for longitudinal and intervention trials. The JANIT Forum will not only validate established NITs but also explore novel NITs and/or combinations of them under the guidance of statistical experts. Innovative NITs will facilitate the selection of the right patients for clinical trials and improve the identification of patients at risk for NASH (fibrosis stages ≥ 2 and NAS ≥ 4) and access to care in clinical settings. The JANIT Forum will continue to educate patient associations and the public about NITs to expand public knowledge of NASH/NAFLD.

## Conclusion

Based on this SWOT analysis, the JANIT Forum aims to develop effective NITs to select patients in the high-risk group of NAFLD patients (those with a high NAS and advanced fibrosis) for HCC surveillance/therapeutic intervention and to determine the effectiveness of therapeutic interventions. The developed NITs will be beneficial for the increasing number of patients with NAFLD as it will allow us to determine the severity of NAFLD and the efficacy of treatment without resorting to liver biopsy.

## Abbreviations

NAFLD: Nonalcoholic fatty liver disease
NASH: Nonalcoholic steatohepatitis
HCC: Hepatocellular carcinoma
NIT: Noninvasive test
FIB-4: Fibrosis-4 index
NFS: NAFLD fibrosis score
ELF test: Enhanced liver fibrosis test
M2BPGi: Mac-2 binding protein glycosylation isomer
ATX: Autotaxin
T4C7S: Type IV collagen 7S
HA: Hyaluronic acid
MRI: Magnetic resonance imaging
CVD: Cardiovascular disease
SWOT: Strengths, weaknesses, opportunities, and threats
JANIT: Japan NASH NIT
AST: Aspartate aminotransferase
ALT: Alanine aminotransferase
APRI: AST to platelet ratio index
BMI: Body mass index
BARD: BMI, AST/ALT ratio, diabetes
T2D: Type 2 diabetes mellitus
AUC: Area under the curve
RIA: Radio immunoassay
CLEIA: Chemiluminescent enzyme immunoassay
M2BP: Mac-2 (galectin-3) binding protein
CHC: Chronic hepatitis type C
CK18-F: Cytokeratin 18 fragment
ELISA: Enzyme-linked immunosorbent assay
LSM: Liver stiffness measurement
VCTE: Vibration-controlled transient elastography
p-SWE: Point shear wave elastography
2D-SWE: 2-Dimensional SWE
MRE: MR elastography
CAP: Controlled attenuation parameter
FDA: US Food and Drug Administration
PH: Portal hypertension
SSM: Spleen stiffness measurement
FAST: FibroScan-AST
ARFI: Acoustic radiation force impulses
SWV: Shear wave velocity
ROI: Region of interest
PDFF: Proton density fat fraction
AI: Artificial intelligence
EHR: Electronic health record
PEST: Politics, Economy, Society, Technology
